# Absence of tmRNA Increases the Persistence to Cefotaxime and the Intercellular Accumulation of Metabolite GlcNAc in *Aeromonas veronii*

**DOI:** 10.3389/fcimb.2020.00044

**Published:** 2020-02-28

**Authors:** Wenjing Yu, Daiyu Li, Hong Li, Yanqiong Tang, Hongqian Tang, Xiang Ma, Zhu Liu

**Affiliations:** ^1^Key Laboratory of Tropical Biological Resources of Ministry of Education, School of Life and Pharmaceutical Sciences, Hainan University, Haikou, China; ^2^School of Tropical Crops, Hainan University, Haikou, China

**Keywords:** *Aeromonas veronii*, β-lactams, persisters, GlcNAc, peptidoglycan biosynthesis

## Abstract

Bacterial persisters are a small proportion of phenotypically heterogeneous variants with the transient capability to survive in high concentrations of antibiotics, causing recurrent infections in both human and aquatic animals. Transfer-messenger RNA (tmRNA), which was encoded by the *ssrA* gene, was identified as a determinant regulator mediating the persistence to β-lactams in the pathogenic *Aeromonas veronii* C4. The deletion of tmRNA exhibited the increased ability of persister formation most probably due to the reduction of protein synthesis. Transcriptomic and metabolomic analyses revealed that the absence of tmRNA not only significantly elevated the intercellular levels of metabolite GlcNAc and promoted NaCl osmotic tolerance, but also upregulated the expression of metabolic genes in both the upstream biosynthesis pathway and the downstream metabolic flux of peptidoglycan (PG) biosynthesis. Finally, exogenous GlcNAc stimulated significant bacterial growth, enhanced content of GlcNAc in the cell wall, higher resistance to osmotic response, and higher persistence to cefotaxime in a concentration-dependent manner, implying its potential role in promoting the multiple phenotypes observed in tmRNA deletion strains. Taken together, these results hint at a potential mechanism of persister formation mediated by tmRNA against the β-lactam challenges in *A. veronii*.

## Introduction

When treated with a lethal concentration of antibiotic, the great majority of bacteria are killed, whereas a small proportion of persisters survive in a dormant state (Fisher et al., 2017). Persister cells never exhibit genetic mutations and thus recover to normal growth and sensitivity after the removal of antibiotics (Helaine and Kugelberg, 2014). Bacterial persistence exists in nearly all bacterial pathogens and acts as the major contributor to the emergence of antibiotic resistance and the relapse of many chronic infectious diseases affecting humans and aquaculture (Michiels et al., 2016).

Despite intensive attempts, the underlying mechanism of persister cell formation is still elusive. It has been reported that bacterial persister formation is associated with trans-translation (Li et al., 2013; Liu et al., 2017), toxin–antitoxin modules (TA modules) (Li and Zhang, 2007), SOS response (Dorr et al., 2009), stringent response (Liu et al., 2017), transporter or efflux mechanism (Pu et al., 2016), and metabolic pathways (Amato et al., 2013). Trans-translation is mediated by transfer-messenger RNA (tmRNA) and small protein B (SmpB) (de la Cruz and Vioque, 2001). It is a vital quality control system for rescuing stalled ribosomes on defective mRNAs (Keiler, 2008). Besides, persister formation can be induced by glucose metabolism and stringent stress via inhibiting peptidoglycan biosynthesis (PGB) in the challenge of β-lactams (Amato and Brynildsen, 2015). Peptide polysaccharide is the final product of PGB and the major component of the bacterial envelope. It is composed of *N*-acetylglucosamine (GlcNAc) and *N*-acetylmuramic acid (MurNAc) bridged by β-1,4 glycosidic bonds (Johnson et al., 2013). Establishing a solid relationship between PGB or trans-translation with persisters production is urgent for both the clinical treatment of chronic infections and aquaculture development (Girgis et al., 2012).

The gram-negative bacterium *Aeromonas veronii* (*A. veronii*) is one of the most threatening pathogens in aquaculture, which initiates massive mortalities in fish species and causes catastrophic economic losses in the fish-farming industry (Li et al., 2011; Reyes-Becerril et al., 2015). *A. veronii* exhibits natural resistance to multiple antibiotics, including sulfonamides and ampicillin (Liu et al., 2018). Deficiency of trans-translation in *Escherichia coli* significantly enhances the sensitivity to aminoglycosides (Luidalepp et al., 2005), while the deletion of tmRNA leads to strong resistance to the β-lactam antibiotics cefotaxime in *A. veronii*. Herein, we explored the role of tmRNA in bacterial persister formation of *A. veronii* against a representative of β-lactam cefotaxime. The results suggested that the absence of tmRNA increased persister formation of *A. veronii* C4 against cefotaxime through a potential mechanism involved in primarily the reduction of protein synthesis, and partly the accumulation of intercellular abundance of elementary metabolite GlcNAc. The results illustrated that trans-translation acted as the critical mediator to connect the carbon resource metabolism with persister formation by changing the levels of an intracellular metabolite, and thereby uncovered a novel model of persistence formation against cefotaxime.

## Materials and Methods

### Bacterial Strains and Growth Conditions

The tmRNA deletion strain of *A. veronii* C4 was constructed previously (Liu et al., 2015). Bacterial culture was shaken (150 rpm) at 30°C for 16 h in LB medium, or grown on LB agar plate at 30°C for 24 h. M9 or LB medium was supplemented with 50 μg/ml ampicillin. Different concentrations of GlcNAc (5, 10, 15, and 20 mM) were added to the media for research purposes.

### The Growth Curve Measurement

Bacteria were cultured for 36 h at the initial turbidity of 0.02 optical densities (OD) per milliliter, and the OD values were measured at 600 nm wavelength with 2-h intervals.

### Minimum Inhibitory Concentration and Minimum Bactericidal Concentration (MBC) Assays

The culture was inoculated into a 96-well-plate with the initial 0.005 OD per milliliter and grown at 30°C for 22 h. The serial two-fold dilutions were performed with the additions of cefotaxime. Three replicates were performed for each strain of bacteria. The MIC values were determined as the lowest antibiotic concentration that inhibited visible growth, and the MBC values were defined as the lowest concentration that killed 99.9% of the bacteria (Liu et al., 2018).

### Persister Assay

Persistence was determined by evaluating the viable bacteria per 1 ml. After shaking at 30°C in M9 or LB medium overnight, bacterial cultures were collected and washed, and then transferred to M9 or LB medium containing 5 μg/ml cefotaxime with initial 1.8 × 10^7^ cells per ml. To test the contribution of GlcNAc to persister formation, the cells were cultured in the presence of 20 mM GlcNAc, washed, and then challenged with 5 μg/ml cefotaxime at 30°C for 11 h. To eliminate the effect of trans-translation malfunction, 50 μg/ml chloramphenicol was added as a protein synthesis inhibitor. The volume of 1 ml culture was collected and serially diluted in phosphate-buffered saline (PBS), and 50 μl of which was plated onto LB agar. The colony-forming units (CFUs) were counted after 22 h incubation at 30°C (Zhang et al., 2019).

### RNA Sequencing and Bioinformatics Analysis

Bacterial strains were cultured in 10 ml of M9 medium containing 50 μg/ml ampicillin at 30°C, shaking at 150 rpm for 20 h. The cells were collected and total RNA was extracted using the traditional phenol/chloroform method. After determining the concentration and quality of the extracted RNA using Agilent 2100 Bio analyzer, the RNA sample was treated with DNase I to remove DNA, followed by the depletion of rRNA with the RiboZero Magnetic Kit. Then, the resulting RNA was used as the template for generating the double-stranded cDNA in a reverse transcription reaction. The synthesized cDNA was subjected to repair and adenylated in the 3′-end and ligated to the adapters. The cDNA fragments were enriched by PCR amplification with a PCR Primer Cocktail, and the purified products were sequenced using a Hiseq Xten (Illumina, San Diego, CA, USA), and 50 bp single-end RNA-seq reads were obtained. Sequence files were generated in the FASTQ format. The RNA-seq raw data were assembled and analyzed in comparison to the translational region of the annotated DNA sequence in reference genomes (GCA_001593245.1 and GCA_000204115.1) using HISAT (Kim et al., 2015). To identify the differential expression between wild type and tmRNA deletion, Bowtie 2(v2.2.5) was used to analyze the mRNA expression (Langmead and Salzberg, 2012). The unit of measurement is fragmented per kilobase of per million fragments mapped (FPKM). It was measured to normalize the gene expression levels, and the *p* value was evaluated for each gene, and Benjamini–Hochberg false discovery rate (FDR) was applied for the correction. Only the comparisons with “FDR” <0.05 and expression fold change greater than two-fold in the Cuffdiff output were represented as significant differential expression. GEO accession number is GSE120603, the URL of the accession website is https://submit.ncbi.nlm.nih.gov/subs/sra/SUB6133286. The DESeq 2 packages in R was applied to estimate the fold changes and to perform other analysis (Love et al., 2014).

### Metabolomic Analysis

The bacteria were cultured for 20 h and were collected and lysed using 1 ml extraction solvent (volume ratios of methanol and acetonitrile and water were 2:2:1, stored at −20°C before use). The extracts were homogenized in ball mill for 4 min, and subjected to ultrasonic treatments in ice water three times, followed by incubating at −20°C for 1 h to precipitate the proteins. After centrifugation at 12,000 rpm for 15 min at 4°C, the supernatant was transferred into EP tubes and dried in a vacuum concentrator. Then, the extraction solvent (volume ratios of acetonitrile and water were 1:1) was added for reconstitution. After vortex and ultrasonication for 10 min at 4°C in the water bath, the reconstituted extracts were spun down, and the supernatant was transferred into an LC/MS glass vial. A total of 10 μl supernatant was taken from each sample and pooled as QC samples, from which 60 μl was used for the UHPLC-QTOF-MS analysis. LC-MS/MS analyses were performed using a UHPLC system (1290, Agilent Technologies) with a UPLC BEH Amide column (1.7 μm 2.1^*^100 mm, Waters) coupled to TripleTOF 6600 (Q-TOF, AB Sciex). AB 6600 Triple TOF mass spectrometer can collect primary and secondary mass spectrometer data based on the IDA function under the control of Analyst TF 1.7 and AB Sciex. In each data acquisition cycle, the molecular ions with the strongest strength >100 were selected as the corresponding secondary mass spectrometry data. MS raw data (.wiff) files were converted to the mzXML format using ProteoWizard and processed by R package XCMS (version 3.2). The preprocessing results generated a data matrix that consisted of the retention time (RT), mass-to-charge ratio (m/z) values, and peak intensity. R package CAMERA was used for peak annotation after XCMS data processing. In-house MS2 database was applied in metabolite identification. The website of metabolomics raw data was accessed at https://www.ebi.ac.uk/metabolights/MTBLS1191.

### Determination of the Relative Content of GlcNAc

The isolation of cell walls was performed according to a previous protocol (Glauner et al., 1988). To compare the content of GlcNAc, the bacteria were harvested and counted, and a total of 1.2 × 10^11^ cells were resuspended in PBS (pH = 7.4) supplemented with 5% SDS for boiling. The pellet was dissolved in PBS appending with 300 μg/ml of α-trypsin shaking overnight at 37°C, followed by lysing with additional 300 μg/ml α-trypsin for 4 h, and incubated in a final concentration of 1% SDS for 1 h at 95°C. After repeated washes to remove SDS, the peptidoglycan was suspended and hydrolyzed with 6 mol/L HCl in boiling water. The relative content of GlcNAc in peptidoglycan was calculated as follows: Relative content of GlcNAc = mass of GlcNAc/mass of peptidoglycan (Zhang and Wu, 1990).

### Measurement of the Response to NaCl Osmotic Stress

To compare the tolerance under NaCl osmotic stress, the bacteria were cultured with initial 1.8 × 10^6^ cells/ml overnight, and a total of 1.8 × 10^8^ cells were collected by centrifugation and resuspended in 1 ml of 2 mol/L NaCl solutions. After culturing at 30°C for 1 h, the suspension was diluted with sterile water to form a series of NaCl gradients, 4 μl of which was plated onto LB solid medium and cultured at 30°C for about 16 h. The colony-forming units (CFUs) were counted after 22 h incubation at 30°C.

### Statistical Analysis

The data were presented as the mean plus standard deviation, and the differences were analyzed with a one-way analysis of variance (ANOVA).

## Results

### The Absence of tmRNA May Increase Persister Formation Under Cefotaxime by Reduction of Protein Synthesis

**Figure 1:**
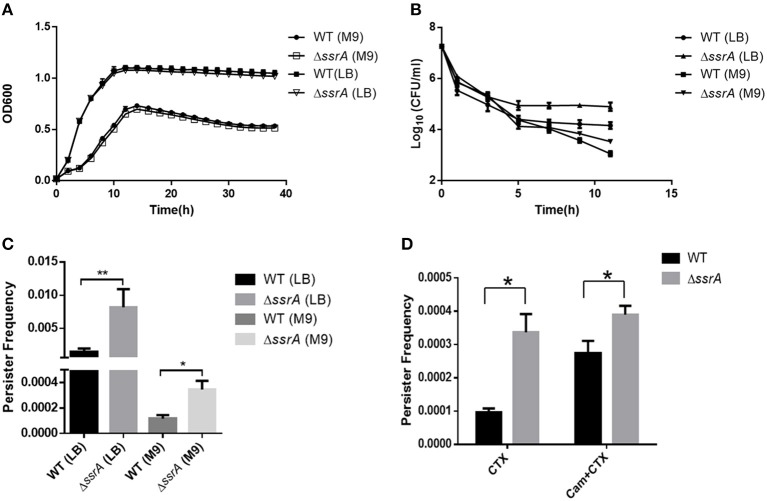
The deletion of tmRNA increases persister formation under cefotaxime treatment. **(A)** Growth curves of wild type (WT) and tmRNA deletion strain (Δ*ssrA*) in LB and M9 medium. **(B)** Bacterial survival of WT and Δ*ssrA* in LB and M9 medium after treatment with cefotaxime for 11 h. *a significant difference with *p* < 0.05, and **an extremely significant result with *p* < 0.01, followed by one-way ANOVA, Tukey post-test. **(C)** Persister frequency of WT and Δ*ssrA* in LB and M9 medium after treatment with cefotaxime for 11 h, wherein persister frequency = CFU post-cefotaxime treatment/CFU pre-cefotaxime treatment. **(D)** Persister frequency of WT and Δ*ssrA* in M9 medium under the treatment of 5 μg/ml cefotaxime (CTX) for 11 h, in the absence or presence of the pretreatment with 50 μg/ml chloramphenicol (Balaban et al., 2019) to inhibit the protein synthesis. *A significant difference with *p* < 0.05, followed by one-way ANOVA, Tukey post-test.

Both the growth curve and the tolerance against cefotaxime were determined in the deletion of *ssrA* strain and the wild type. The results showed that the deletion of *ssrA* did not change the growth rate of *A. veronii* C4 in LB or M9 media, while both the deletion of *ssrA* and the wild type grew slower in M9 media because of the lack of nutrients (Figure 1A and Figures S1A,B). The MIC values of cefotaxime for both wild type and *ssrA* mutants were 0.05 μg/ml, and the MBC values for both were 2 μg/ml, indicating that *A. veronii* C4 was sensitive to cefotaxime and the deletion of *ssrA* did not affect the susceptibility against cefotaxime. Although *A. veronii* C4 encoded beta-lactamases including AMS64_18215, AMS64_00580, and AMS64_13110, cefotaxime was inherently resistant to most beta-lactamase enzymes, similarly with the previous report in *E. coli* (Barour et al., 2019). However, the biphasic kill curve revealed that significant differences in persister cell formation were observed in both LB and M9 media between wild type and *ssrA* mutants under the treatment of 5 μg/ml cefotaxime (Figure 1B). Consistently, the enumeration of persister cells showed that the deletion of *ssrA* significantly increased the persister production by five- and four-fold in LB and M9 media (Figure 1C) after incubation with 5 μg/ml cefotaxime for 11 h, respectively. The concentration of 5 μg/ml cefotaxime was selected as the suggested concentration, which should be 10 times the MIC value in persister assay, to distinguish the persistence from transient modes of resistance (Balaban et al., 2019).

To verify whether the promoted persistence was caused by the reduction of the protein synthesis, both wild type and *ssrA* deletion mutants were pretreated with a protein synthesis inhibitor chloramphenicol, and subsequently, the persister formation was determined to challenge with cefotaxime. The results showed that persister frequency was only elevated in wild type rather than tmRNA deletion strains, suggesting that both the absence of *ssrA* and chloramphenicol treatment produced a slow-growing or dormant state through the inhibition of translation (Figure 1D). Moreover, the formations of persister cells were significantly reduced compared to *ssrA* deletion mutants when cells were propagated in LB (Figures 1B,C), likely due to the higher frequency of protein synthesis and more susceptibility to cefotaxime as the results of richer nutrients in LB media (VanHook, 2019). Taken together, these results suggested that the deletion of tmRNA strain might mediate higher persister cell formation under the pressure of cefotaxime by reduction of protein synthesis.

### The Absence of tmRNA Led to the Accumulation of Metabolite GlcNAc

**Figure 2:**
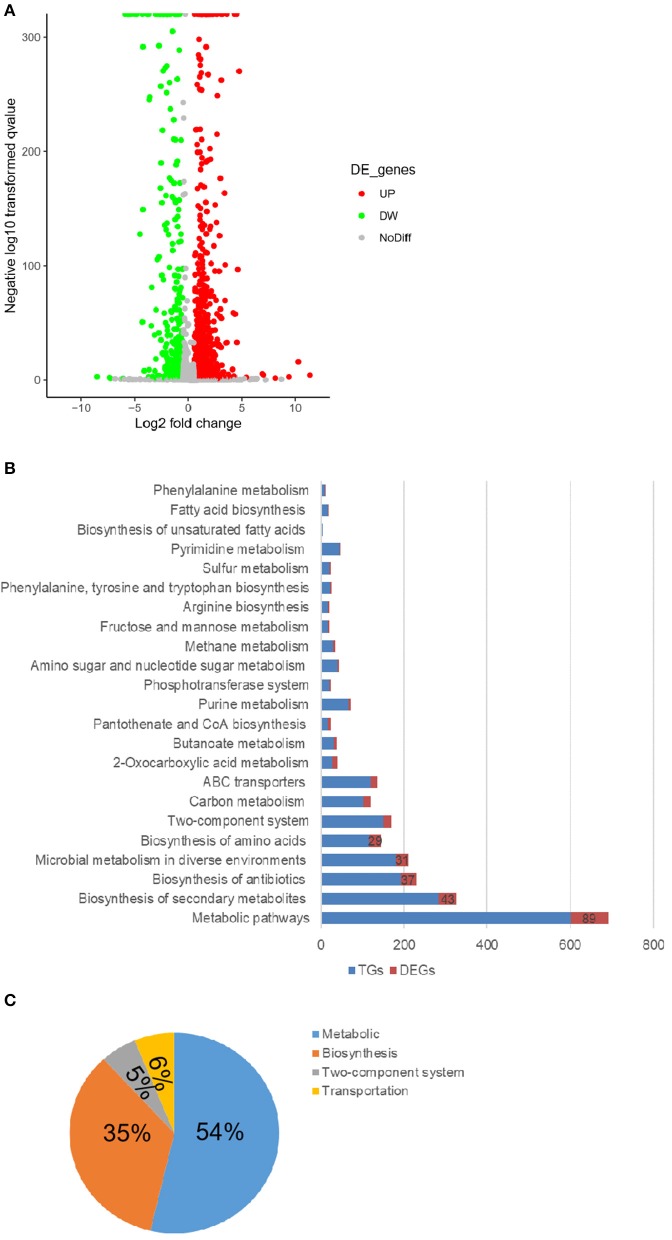
The deletion of tmRNA dominantly changes the metabolic-related genes. **(A)** Volcanic map showing the changes of individual genes in WT and Δ*ssrA* strains, wherein the red circles represent the upregulated genes (UP), and green circles represent the downregulated genes (DW) in Δ*ssrA* strain, which exhibit at least two-fold changes with *p* < 0.05. Gray circles represent the genes without expression differences (NoDiff). **(B)** Enrichment of genes with differential changes in the KEGG pathway, wherein the blue bars represent the number of all the detected genes in the pathway (TGs), and red bars represent the number of the genes with significant changes (DEGs). **(C)** The primary classification of the altered genes in the KEGG pathway in terms of their basic functions, wherein the blue portion represents the metabolic-related pathway, the orange portion represents the synthetic-related pathway, the gray portion represents the two-component system, and the yellow portion represents the transport-related pathway.

To explore the genes and pathways responsible for the effects of tmRNA on persister production, the transcriptional differences were compared between wild type and *ssrA* deletion strains. The results showed that 47% of the total detected genes exhibited altered expression levels in the *ssrA* deletion strain, wherein 36% increased and 11% decreased (Figure 2A). KEGG pathway enrichment indicated that the genes with varied expression levels were mostly involved in metabolic pathways and biosynthesis of secondary metabolites, with significant changes of 89 and 43 genes, respectively (Figure 2B). The metabolic-related genes constituted the most dominant portion, occupying 54% of the total genes with significant changes, followed by the biosynthesis-related genes that shared 35% proportion (Figure 2C). These results implied that the deletion of *ssrA* in *A. veronii* C4 primarily influenced the metabolism- and biosynthesis-related genes.

**Figure 3:**
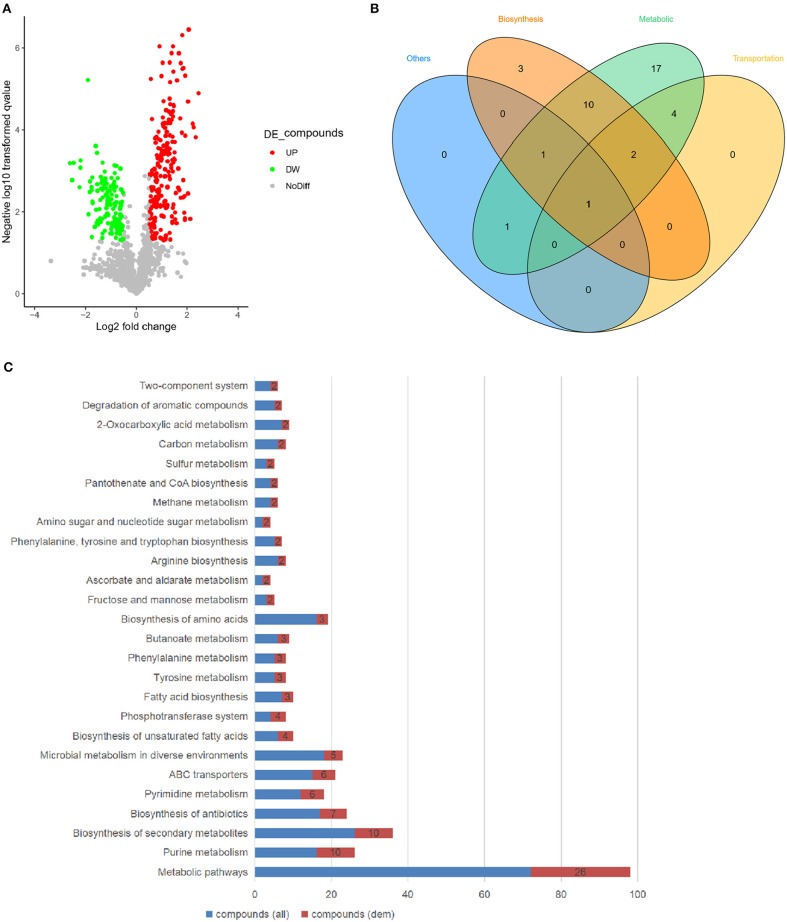
The deletion of tmRNA significantly upregulates the level of the metabolite GlcNAc. **(A)** Volcanic map showing changes of essential metabolites in WT and Δ*ssrA* strains, wherein the red circles represent the metabolites with increased abundance (UP), and green circles represent the metabolites with reduced abundance (DW) in Δ*ssrA* strain, which exhibit at least two-fold changes with *p* < 0.05. The gray circles represent the metabolites without differences in abundance (NoDiff). **(B)** Venn graph showing the unique and overlapping relationships between the altered metabolites in terms of their basic classifications of functions, with the blue portion representing other pathways, the orange portion representing synthetically related pathways, the green portion representing metabolically related pathways, and the yellow portion representing transport-related pathways. **(C)** Enrichment of the differential metabolites in the KEGG pathway, wherein the blue bars represent the number of all the detected compounds in the pathway [compound (all)] and the red bars represent the number of the detected compounds with changes [compound (dem)].

Accordingly, the variations of the essential metabolites were compared between wild type and *ssrA* deletion strains through untargeted metabolomic analysis using UHPLC-QTOF-MS, which identified the primary metabolites essential for bacteria growth, development, and reproduction of bacteria (Sanchez and Demain, 2008). The results showed that 13% of the total detected metabolites exhibited higher abundances in the *ssrA* deletion strain, while 8 and 79% of which were found to have lower or unchanged abundances, separately (Figure 3A). The primarily differential metabolites included organic nitrogen compounds, carboxylic acids and derivatives, benzene and substituted derivatives, phenols, etc. (Table S1). Primary functional classification in the Venn diagram illustrated that the metabolic group occupied the highest numbers of unique metabolites, followed by the biosynthesis group (Figure 3B). This result is consistent with that found by transcriptomic analysis (Figure 2C). Further KEGG pathway analysis demonstrated that the alterations of metabolic pathways contributed dominantly, with 26 compounds changed, followed by purine metabolism, pyrimidine metabolism, and fructose and mannose metabolism, etc. (Figure 3C). Considering that the antibiotic cefotaxime takes actions by inhibiting the activity of peptidoglycan transpeptidase and thus the bacterial cell wall synthesis, which results in cell lysis and death (Lefrock et al., 2012), we emphasized the amino sugar and nucleoside sugar metabolism pathways and discovered that two metabolites, i.e., GlcNAc (C00140 in Table S2) and L-arabinose (C00259 in Table S2), exhibited higher abundance in the *ssrA* deletion strain. Besides the amino sugar and nucleoside sugar metabolism, GlcNAc functions in phosphotransferase system (PTS) and ABC transporters, while L-arabinose participates in ABC transporters, ascorbate, and aldarate metabolism (Table S2). GlcNAc exhibited a higher abundance level than L-arabinose in the *ssrA* deletion strain (Figure S2 and Table S3).

**Figure 4:**
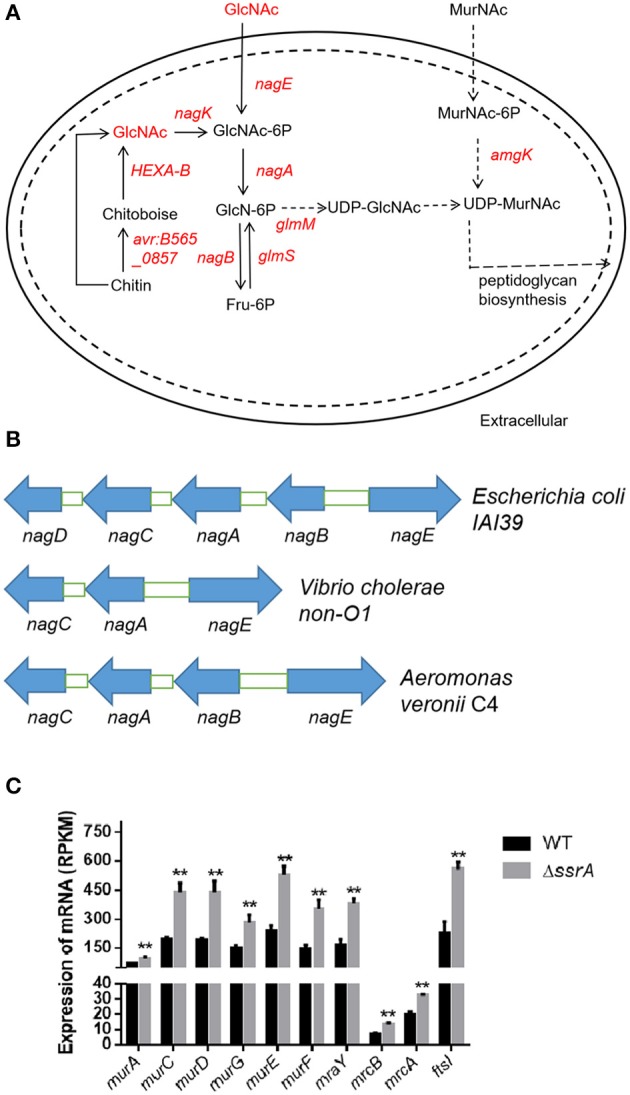
The deletion of tmRNA enhances the expression of genes associated with the metabolism of GlcNAc. **(A)** Intercellular amino sugar metabolic pathways involved by GlcNAc. The significantly upregulated genes and metabolites are shown in red, and those with no significant differences are shown in black. The arrows indicate the direction of the metabolic flux. **(B)** The genetic locations of *nagE, nagA, nagB, nagC*, and *nagD* on the chromosome in *Escherichia coli IAI39, Vibrio cholerae non-O1*, and *Aeromonas veronii* C4. Blue arrows indicate the transcriptional directions of the genes. **(C)** The gene expression levels of peptidoglycan synthesis. **An extremely significant result with *p* < 0.01, followed by one-way ANOVA, Tukey post-test.

We investigated the GlcNAc-involved pathways and found further that GlcNAc is a preliminary substrate to form the precursor UDP-GlcNAc for peptidoglycan biosynthesis (Figure 4A). The transcriptional data revealed that the upregulation of *HEXA_B* might contribute to the increase of GlcNAc. Besides, enhanced expression of the *nag* operon may stimulate the formation of UDP-GlcNAc (the regulators in red in Figure 4A and Figure S3A). The *nag* operon in *A. veronii* C4 was composed of the genes *nagE, nagB, nagA*, and *nagC*, which were located adjacently on the chromosome and arranged similarly in other bacterial species such as *E. coli* and *Vibrio cholerae non-O1* (Figure 4B). The phylogenetic analysis revealed that each gene in the *nag* operon was clustered into the genus *Aeromonas*, implying that they may exhibit the same functionality (Figure S3B). The heightened expression of *nagE* and *nagK* probably promoted the phosphorylation of GlcNAc for the formation of GlcNAc-6p, subsequently transforming GlcNAc-6p to glucosamine-6-phosphate (GlcN-6p) by elevated expression of *nagA*, and eventually producing fructose-6-phosphate (Fru-6p) by upregulated *nagB* (Figure 4A). In addition, the enhanced expression of the *amgK* gene facilitated the formation of UDP-MurNAc (Figure 4A). As for the downstream pathways, the transcription levels of the genes involved peptidoglycan synthesis were investigated (Figure S3C). The related genes were categorized into two subgroups encoding Mur ligases and penicillin-binding proteins (PBPs), both of which function in peptidoglycan biosynthesis and the assembly of the bacterial cell wall. The Mur ligases (*murC, murD, murE*, and *murF*) were significantly upregulated in the *ssrA* deletion mutant. Similarly, so did PBPs (*mrcB, mrcA, ftsI, mrdA*, and *pbpC*) (Figure 4C and Figures S3C,D). They are attractive targets for designing novel antibacterial agents to inhibit cell wall synthesis (Barreteau et al., 2010; Welsh et al., 2017). In particular, the high-molecular-weight PBPs (*mrcB, mrcA*, and *ftsI*) have been identified as the targets of cefotaxime, which catalyze the synthesis of the major component of the cell wall (Denome et al., 1999).

**Figure 5:**
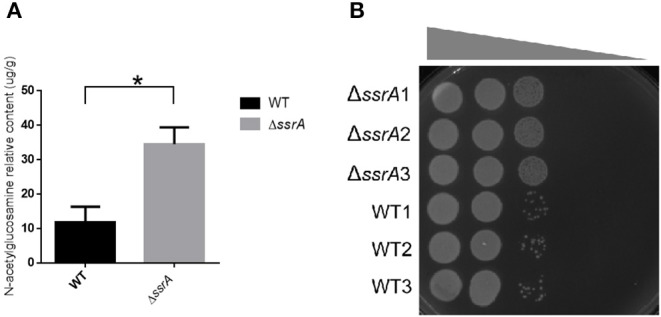
The deletion of tmRNA promotes the peptidoglycan biosynthesis. **(A)** The relative contents of GlcNAc in the peptidoglycan of the cell wall in WT and Δ*ssrA* strains. *A significant difference with *p* < 0.05, followed by one-way ANOVA, Tukey post-test. **(B)** The response of WT and Δ*ssrA* strains to NaCl osmotic stress. An identical number of initial bacteria (2.88 × 10^4^ cells) were cultured in the growth medium containing a series of NaCl gradients. The gray wedge indicates that the osmotic stress decreases from left to right.

In order to validate the effects of the accumulation of cellular GlcNAc, we extracted the peptidoglycans and determined the content of GlcNAc in wild type and *ssrA* deletion strains. Consistent with the transcriptional data, the results showed that the GlcNAc content in the peptidoglycan of the deletion strain was significantly higher than that of the wild type (Figure 5A). Moreover, considering that the enhanced peptidoglycan usually leads to improved tolerance to osmotic stress (Vollmer et al., 2008), we determined the responses of wild type and *ssrA* deletion strains to NaCl osmotic stress. The results showed that the *ssrA* deletion strain exhibited remarkably enhanced tolerance in comparison to the wild type (Figure 5B). Taken together, these results confirmed that the tmRNA deletion strain increases the intercellular stores of metabolite GlcNAc.

### Exogenous GlcNAc Promotes the Multiple Phenotypes as Observed in tmRNA Deletion Strains

**Figure 6:**
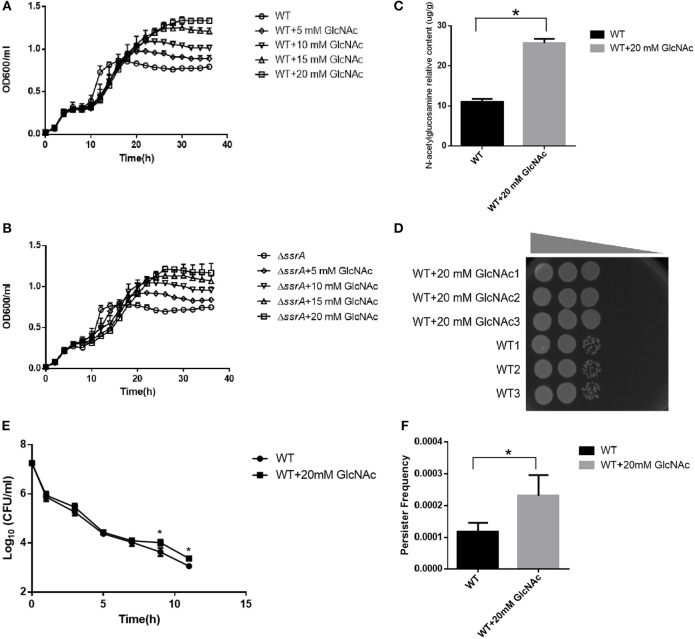
Metabolites GlcNAc promotes the peptidoglycan biosynthesis and the persister formation. **(A,B)** Growth curves of WT and Δ*ssrA* strain supplemented without and (WT/Δ*ssrA*) with 5 mM GlcNAc (5 mM GlcNAc + WT/Δ*ssrA*), 10 mM GlcNAc (10 mM GlcNAc + WT/Δ*ssrA*), 15 mM GlcNAc (15 mM GlcNAc + WT/Δ*ssrA*), and 20 mM GlcNAc (20 mM GlcNAc + WT/Δ*ssrA*) in M9 medium. **(C)** The relative contents of GlcNAc in the peptidoglycan of the cell wall in WT supplemented without or with 20 mM GlcNAc, *a significant difference with *p* < 0.05, followed by one-way ANOVA, Tukey post-test. **(D)** The bacterial response to NaCl osmotic stress. An identical number of initial bacteria (2.88 × 10^4^ cells) were adopted in the osmotic response experiment, with the gray wedge indicating that the osmotic stress decreases from left to right. **(E,F)** Bacterial survival curve and persister frequency showing the persister formations in WT in the absence and presence of 20 mM GlcNAc in M9 medium under the treatment with cefotaxime for 11 h. *A significant result with *p* < 0.05, followed by one-way ANOVA, Tukey post-test.

In order to validate the contributions of the accumulated GlcNAc for the multiple phenotypes observed in the *ssrA* deletion strain, different concentrations of exogenous GlcNAc were added to wild type of *A. veronii* C4, followed by evaluations of the bacterial growth, persister formations, GlcNAc contents in peptidoglycan, and response to osmotic stress. The growth curve demonstrated that addition of GlcNAc caused faster growth rates and higher cell mass at stationary phase in a concentration-dependent manner, while there were no significant growth differences at the exponential phase in both wild type and *ssrA* deletion mutants (Figures 6A,B and Figures S4A,B). Moreover, accompanied by an increased content of GlcNAc in the cell wall (Figure 6C), the resistance to NaCl osmotic stress was enhanced (Figure 6D). These results illustrated that GlcNAc acted as the major contributor for the enhanced peptidoglycan and resistance to osmotic stress in the *ssrA* deletion strain. In addition, when the wild type was treated with 5 μg/ml cefotaxime in M9 medium, persister production was doubled in the presence of 20 mM of GlcNAc in both the biphasic kill curve and persister cell enumeration assay (Figures 6E,F). Similar results were displayed in the LB medium Figures S4C,D). We proposed that the additive of GlcNAc may contribute in part to persister cell formation. These results illustrated that enhanced GlcNAc levels may account for the phenotypes associated with the deletion of *ssrA* strain.

## Discussion

The trans-translation system is required for antibiotic tolerance, growth, and persistence under antibiotic and environmental stresses (Keiler, 2008). In this study, the role of tmRNA, encoded by the *ssrA* gene as one of the core factors in the trans-translation system, in persister formation was investigated in the aquatic pathogen *A. veronii* C4. Our results showed that the *ssrA* deletion mutant had a growth rate identical to that of wild type (Figure 1A and Figures S1A,B), indicating that the tmRNA was dispensable to bacterial growth in *A. veronii* C4. The same result was reported for some other bacterial species (de la Cruz and Vioque, 2001). However, the results surprisingly showed that the deletion of *ssrA* promoted significantly higher persister production under the treatment of cefotaxime (Figures 1B,C), although it did not cause different MIC/MBC values as compared with wild type. Although the deletion of *ssrA* strain promotes higher susceptibility to fosfomycin and ampicillin (Luidalepp et al., 2005), it does not significantly affect responses to ampicillin (de la Cruz and Vioque, 2001). In *Streptococcus pneumoniae*, tmRNA deficiency may reduce ROS production and chromosome breakage through the trans-translation process, thus increasing the tolerance to fluoroquinolones (Liliana et al., 2016).

Herein, for the first time, the effect of the *ssrA* mutant was delineated on the persistence to cefotaxime. Our results suggested that the inhibition of translation probably constituted the primary reason for the boost of persister cell formation in the presence of cefotaxime (Figure 1D). Although chloramphenicol treatment led to the lower translation frequency and prompted a slow-growing or dormant state, there was still a slight difference in the persistence compare to in *ssrA* deletion strains (Figure 1D). Further, the transcriptomic and metabolomic analyses were conducted to identify the related genes and pathways. By analyzing the transcriptomic profile, the absence of tmRNA elevated the expressions of the key enzymes in the metabolic and the biosynthesis pathways (Figures 2A–C). Besides, metabolomic analyses indicated that the *ssrA* deletion significantly increased the abundance of metabolite GlcNAc, which was involved in the amino sugar and nucleotide sugar metabolism (Figure 3C and Table S3). GlcNAc is an excellent energy source for gram-negative pathogenic bacteria, which can be utilized more efficiently than glucose in multiple metabolic pathways (Hsing-Chen et al., 2002). The supplementation with GlcNAc significantly promoted the growth rate of wild type (Figures 6A,B), including increased relative content of GlcNA in the cell wall (Figure 6C) and higher resistance to osmotic stress (Figure 6D), and promoted persister formation under cefotaxime (Figures 6E,F).

Taken together, the persister formation may be modulated by multiple pathways under cefotaxime in *A. veronii*. First, the reduction of protein synthesis likely acted as the primary contributor (Figure 1D). Secondly, the accumulation of metabolite GlcNAc also promoted the persister formation, probably through accelerating the bacteria growth (Figures 6A,B,E,F). Finally, the upregulation of PBPs in the *ssrA* mutant (Figure S3C) offered a potential explanation for the enhanced tolerance to cefotaxime, probably by providing more target enzymes of cefotaxime (Fani et al., 2013). Although the inhibition of trans-translation by the deletion of *ssrA* caused the depletion of some ribosomes, the reasonable explanation was that the upregulated transcription of PBPs promoted the production. Collectively, these three alternative ways were responsible for persistence. However, which way was dominant depended on the triggering of circumstance effects, and the underlying mechanism needed to be further investigated.

Both trans-translation and metabolism play critical roles in the process of persistence (Li et al., 2013; Amato et al., 2014). The malfunction of SmpB or tmRNA modulates the effector (p)ppGpp to obstruct the persister formation in the presence of aminoglycosides, quinolones, and β-lactams (Liu et al., 2017), and SmpB knockout reduces the number of persisters under gentamicin stress (Liu et al., 2018). Moreover, metabolic inactivity increases persistence (Girgis et al., 2012). Although tmRNA regulates the metabolism of some sugars through the mechanism of trans-translation (Roche and Sauer, 2001), the interactive relationship between trans-translation and metabolism in persister formation remains unclear. The alternations of metabolic flux stimulate the persister formation against fluoroquinolone in a manner of TA behavior by a metabolite–enzyme interaction model, in which the metabolite ppGpp acts as a toxin, and the ppGpp hydrolase SpoT acts as an antitoxin (Amato et al., 2013; Maisonneuve and Gerdes, 2014). Besides, the glucose exhaustion stimulates persister formation to β-lactams through the action of the metabolite ppGpp, mediated by the actions of trans-translation (Amato and Brynildsen, 2015). Dissimilar types of antibiotics may stimulate diverse metabolite–enzyme interaction models under the regulation and control of various mechanisms, thereby inducing different persister levels. Our present study was preliminarily presumed that carbon metabolism was associated with persister formation to β-lactams through the action of the metabolite GlcNAc, which exhibits similar function to ppGpp. Although Mur ligases and penicillin-binding proteins (PBPs) were upregulated and utilized for peptidoglycan biosynthesis and the assembly of the bacterial cell wall, GlcNAc was accumulated curiously. Our explanations assume that GlcNAc participates in various metabolic pathways, and some other metabolic pathways will be downregulated. The contributors that reduce the utilization of GlcNAc remain to be investigated in the *ssrA* mutant. The targeted enzymes that interact with GlcNAc for mediating the persister formation will facilitate the discovery of novel therapeutic strategies against the threatening bacterial pathogens.

## Data Availability Statement

The datasets generated for this study can be found in the NCBI: GSE120603, https://submit.ncbi.nlm.nih.gov/subs/sra/SUB6133286, Metabolights: https://www.ebi.ac.uk/metabolights/MTBLS1191.

## Author Contributions

ZL, XM, and WY contributed to the conception and design of the study. WY and DL performed the experiments. WY, DL, HL, YT, and HT performed the statistical analysis. WY, XM, and ZL drafted the manuscript. All authors contributed to manuscript revision and approved the submitted version.

### Conflict of Interest

The authors declare that the research was conducted in the absence of any commercial or financial relationships that could be construed as a potential conflict of interest.
