# Supplementary material for: Absence of tmRNA Increases the Persistence to Cefotaxime and the Intercellular Accumulation of Metabolite GlcNAc in Aeromonas veronii
Source: Front Cell Infect Microbiol. 2020 Feb 28;10:44. doi: 10.3389/fcimb.2020.00044 (PMC7058587; doi:10.3389/fcimb.2020.00044)
Supplement: Supplementary file 1 [file Data_Sheet_1.pdf]

Supplementary Material

Table S1. Names and classifications of the essential metabolites with altered levels between wild-type and tmRNA deletion strain of *A. veronii* C4

Name of the metabolites	Classification of the metabolites
Uracil	Diazines
4-Guanidinobutyric acid	Organic acids and derivatives
L-Iditol	Organic oxygen compounds
Diacetyl	unknown
L-Glutamate	Carboxylic acids and derivatives
(+-)-Mevalonolactone	unknown
Ethylmalonic acid	Lipids and lipid-like molecules
Triethanolamine	Amines
2-Hydroxyadenine	Organoheterocyclic compounds
Xanthine	Organoheterocyclic compounds
Lavandulol	unknown
Isocaproic acid	Fatty Acyls
DL-Norvaline	unknown
Succinic acid	unknown
Hippuric acid	Benzene and substituted derivatives
Pyruvaldehyde	Organooxygen compounds
Butyl lactate	Carboxylic acids and derivatives
Nicotinate	Pyridines and derivatives
D-Mannitol	Carbohydrates and carbohydrate conjugates
D-Sorbitol	Organic oxygen compounds
Citramalic acid	Fatty Acyls
L-Phenylalanine	Organic acids and derivatives
(R)-3-Hydroxybutyric acid	Organic acids and derivatives

L-Norleucine	Organic acids and derivatives
L-Arginine	Carboxylic acids and derivatives
Ethyl 3-hydroxybutyrate	unknown
L-Citrulline	Organic acids and derivatives
N-Methyl-1-deoxynojirimycin	Piperidines?
Dulcin	unknown
2-Phenylbutyric acid	Benzene and substituted derivatives
4-Hydroxybenzaldehyde	unknown
N-Acetyl-D-glucosamine	Organic oxygen compounds
Val-Ala	unknown
L-Methionine	Organic acids and derivatives
5-Hydroxyindoleacetate	Indoles and derivatives
cis-4-Hydroxy-D-proline	Organic acids and derivatives
Pectin (Galacturonic acid)	Organic oxygen compounds
Agmatine	Organic nitrogen compounds
Glycerol 3-phosphate	Lipids and lipid-like molecules
Dopamine	Phenols
.beta.-Citronellol	unknown
p-CHLOROPHENYLALANINE	unknown
N-Tigloylglycine	unknown
3-Hydroxybenzoate	unknown
2-Hydroxy-3-methoxybenzoic acid	unknown
DL-Phenylalanine	Organic acids and derivatives
N-Acetyl-L-methionine	Organic acids and derivatives
2,2-Dimethyl Succinic acid	unknown
Jasmine lactone	Organoheterocyclic compounds
Val-Glu	Organic acids and derivatives
(R)-(+)-Citronellic acid	Prenol lipids
Leu-Val	unknown
N-Carboxyethyl-.gamma.-aminobutyric acid	unknown
Pro-Val	unknown

sn-Glycerol 3-phosphoethanolamine	Lipids and lipid-like molecules
N-.alpha.-Acetyl-L-arginine	unknown
Val-Val	unknown
Ser-Leu	unknown
Dodecanoic acid	Fatty Acyls
Ala-Glu	unknown
Val-Thr	unknown
Pantothenate	Carboxylic acids and derivatives
Pyrantel	unknown
4-Aminobutyric acid	Organic acids and derivatives
Isovalerylglycine	Organic acids and derivatives
Dimethyl sulfone	Sulfonyls
Nitrotyrosine	unknown
Pro-Ala	unknown
2-Amino-2-methyl-1,3-propanediol	unknown
Leu-Asp	unknown
Val-Ile	unknown
Ile-Thr	unknown
Glu-Ser	unknown
Arg-Val	unknown
Bupropion	Organooxygen compounds
L-Tryptophan	Organoheterocyclic compounds
Lumichrome	unknown
3-Methoxytyramine	Benzenoids
Cytidine	unknown
Uridine	Pyrimidine nucleosides
Pro-Glu	unknown
Pinacidil	unknown
Ile-Ile	unknown
Ile-Asn	unknown
Ser-Val	unknown
Arg-Ala	unknown
Myristic acid	Fatty Acyls
gamma-L-Glutamyl-L-valine	Organic acids and derivatives
Pro-Asn	unknown

Oleic acid	unknown
Val-Met	unknown
Deoxyadenosine	Nucleosides, nucleotides, and alogues
L-NG-Monomethylarginine	unknown
5-Aminopentanoic acid	Organic acids and derivatives
N-Acetyl-L-glutamate	Organic acids and derivatives
Deoxyinosine	Nucleosides, nucleotides, and alogues
Deoxyinosine	Nucleosides, nucleotides, and alogues
5-Amino-4-carbamoylimidazole (AICA)	Organoheterocyclic compounds
3-Hydroxyisovaleric acid	Lipids and lipid-like molecules
Primidone	
Glycerophosphocholine	Lipids and lipid-like molecules
L-Pyroglutamic acid	Organic acids and derivatives
1-Methylpseudouridine	unknown
Ribothymidine	Nucleosides, nucleotides, and alogues
Primaquine	unknown
Ile-Glu	unknown
Ile-Ser	unknown
Pentadecanoic Acid	Lipids and lipid-like molecules
Ser-Asp	unknown
Ile-Met	unknown
L-Asparagine	Organic acids and derivatives
Val-Phe	unknown
trans-Vaccenic acid	Lipids and lipid-like molecules
NG,NG-dimethyl-L-arginine(ADMA)	unknown
Oxprenolol	unknown
Adenosine	Purine nucleosides
Inosine	Nucleosides, nucleotides, and alogues

Acetylcarnitine	unknown
Deoxycytidine	Nucleosides, nucleotides, and alogues
5,2'-O-dimethylcytidine	unknown
beta-Estradiol	Steroids and steroid derivatives
5.alpha.-Androstan-17.beta.-ol-3-one	unknown
Lamivudine	unknown
Val-Arg	unknown
Palmitic acid	Fatty Acyls
Ser-Gln	unknown
Kinetin	unknown
Saccharopine	unknown
Ile-Tyr	unknown
N,N-Dimethylaniline	Organic nitrogen compounds
1-Methyladenosine	Purine nucleosides
3-Ureidopropionate	unknown
Hydroxyisocaproic acid	Lipids and lipid-like molecules
Pelletierine	unknown
Guanosine	Nucleosides, nucleotides, and alogues
His-Glu	unknown
Glycerol 1-myristate	unknown
Ile-Arg	unknown
Heptadecanoic acid	Lipids and lipid-like molecules
D-Ribulose 5-phosphate	Carbohydrates and carbohydrate conjugates
D-Ribulose 5-phosphate	Carbohydrates and carbohydrate conjugates
His-Val	unknown
L-Glutamine	Organic acids and derivatives
Securinine	unknown
Stearidonic Acid	unknown
Phe-Glu	unknown
alpha-Linolenic acid	Fatty Acyls

S-Methyl-5'-thioadenosine	Nucleosides, nucleotides, and alogues
Linoleic acid	unknown
(Z)-6-Octadecenoic acid	unknown
Dehydroabietic acid	unknown
Stearic acid	Fatty Acyls
Ser-Arg	unknown
Arg-Gln	unknown
Arg-Glu	unknown
D-Glucose 6-phosphate	Organic oxygen compounds
Isopentenyl pyrophosphate	Lipids and lipid-like molecules
Triadimefon	unknown
Methoprene (S)	unknown
Tyr-Met	unknown
2'-Deoxyadenosine 5'-monophosphate (dAMP)	Nucleosides, nucleotides, and alogues
Tyr-Asp	unknown
Leu-Trp	unknown
Tyr-Ala	unknown
His-Tyr	unknown
1-methylguanosine	Nucleosides, nucleotides, and alogues
Phe-Arg	unknown
D-Fructose 1,6-bisphosphate	Organic oxygen compounds
Cytidine 5'-monophosphate	unknown
Uridine 5'-monophosphate (UMP)	Nucleosides, nucleotides, and alogues
3-Methoxy-4-Hydroxyphenylglycol Sulfate	Benzenoids
N,N-Dimethylsphingosine	Organic nitrogen compounds
2'-Deoxyinosine	unknown
Phe-Tyr	unknown
Adenosine 2',3'-cyclic monophosphate	unknown
Desipramine	Organoheterocyclic compounds

Arg-Asp	unknown
Tyr-Ser	unknown
Argininosuccinic acid	Organic acids and derivatives
Erucamide	unknown
Tyr-Val	unknown
2'-O-methyladenosine	Nucleosides, nucleotides, and alogues
Phe-Asn	unknown
Stearamide	Fatty Acyls
3',5'-Cyclic guanosine monophosphate	unknown
Pantoprazole	unknown
Adenosine 3'-monophosphate	Nucleosides, nucleotides, and alogues
1-Palmitoylglycerol	unknown
Inosine 5'-monophosphate (IMP)	Nucleosides, nucleotides, and alogues
Adenosine 3',5'-cyclic phosphate (cAMP)	unknown
Homogentisic acid	Benzenoids
Behenic acid	Fatty Acyls
Isomaltose	Carbohydrates and carbohydrate conjugates
Cellobiose	unknown
Ala-Pro	unknown
Ile-Trp	unknown
24,25-Hydroxyvitamin D3	unknown
Dihydrotachysterol	Steroids and steroid derivatives
Arg-Trp	unknown
Cytidine 5'-diphosphate (CDP)	Nucleosides, nucleotides, and alogues
Ala-Thr	unknown
Uridine 5'-diphosphate (UDP)	Nucleosides, nucleotides, and alogues
Thiamine monophosphate	Organoheterocyclic compounds
Normetanephrine	unknown
Cholecalciferol (Vitamin D3)	unknown

Lathosterol	unknown
Adenosine 3',5'-diphosphate (PAP)	Nucleosides, nucleotides, and alogues
alpha-Guanidinoglutaric Acid	unknown
(-)-Tylocrebrine	unknown
Guanosine 5'-diphosphate (GDP)	Nucleosides, nucleotides, and alogues
1-Palmitoyl-2-hydroxy-sn-glycero-3-phosphoethanolamine	unknown
Terfenadine	unknown
Taurolithocholic acid	unknown
cis-9-Palmitoleic acid	Lipids and lipid-like molecules
Vitamin K1	Lipids and lipid-like molecules
Gln-Asn	unknown
Maltotriose	Carbohydrates and carbohydrate conjugates
Testosterone	Steroids and steroid derivatives
Cytidine 2',3'-cyclic phosphate	Nucleosides, nucleotides, and alogues
Stachyose	Organic oxygen compounds
Glycogen	Organic oxygen compounds
Acetyl coenzyme A (Acetyl-CoA)	unknown

Table S2. KEGG pathway affiliation for the essential metabolites with altered levels between wild-type and tmRNA deletion strain of *A. veronii* C4.

Pathway	KEGG COMPOUND database ID for the affiliated metabolites
Metabolic pathways	C00106;C00042;C10164;C00178;C00259;C00262;C00385;C00794;C00079;C01089;C00327;C00140;C00093;C00475;C00299;C06424;C00559;C00624;C00294;C00387;C06427;C00092;C00354;C00130;C00544;C00112
Purine metabolism	C00262;C00385;C00559;C00294;C00387;C00942;C01367;C00130;C00575;C00054
Biosynthesis of secondary metabolites	C00042;C00483;C00385;C00079;C00327;C00093;C00624;C06427;C00354;C00130
Biosynthesis of antibiotics	C00042;C00079;C00327;C00624;C00092;C00354;C00130
Pyrimidine metabolism	C00106;C02376;C00178;C00475;C00299;C00112
ABC transporters	C00259;C00392;C00794;C00079;C00140;C00093
Microbial metabolism in diverse environments	C00042;C05629;C00385;C00354;C00544
Biosynthesis of unsaturated fatty acids	C00712;C06427;C01530;C08281
Phosphotransferase system (PTS)	C00392;C00794;C00140;C00092
Fatty acid biosynthesis	C06424;C00712;C01530
Tyrosine metabolism	C00042;C00483;C00544
Phenylalanine metabolism	C00042;C05629;C00079
Butanoate metabolism	C00042;C00741;C01089
Biosynthesis of amino acids	C00079;C00327;C00624
Fructose and mannose metabolism	C00392;C00794
Ascorbate and aldarate metabolism	C01620;C00259
Arginine biosynthesis	C00327;C00624

Phenylalanine, tyrosine and tryptophan biosynthesis	C00079;C00354
Amino sugar and nucleotide sugar metabolism	C00259;C00140
Methane metabolism	C00483;C00354
Pantothenate and CoA biosynthesis	C00106;C00054
Sulfur metabolism	C00042;C00054
Carbon metabolism	C00042;C00354
2-Oxocarboxylic acid metabolism	C00079;C00624
Degradation of aromatic compounds	C00042;C05629

Table S3. KEGG pathway affiliation for the essential metabolites with significantly altered levels between wild-type and tmRNA deletion strain of *A. veronii* C4.

**Pathway**	**KEGG COMPOUND database ID for the significant differential metabolites**
Purine metabolism	C00130; C00559; C00294; C00385; C00387; C00262; C00575; C01367; C00942
Pyrimidine metabolism	C00299; C00112; C00475; C02376; C00106; C00178
Tyrosine metabolism	C00483; C00042
Phenylalanine metabolism	C05629; C00042; C00079
Biosynthesis of unsaturated fatty acids	C01530
alpha-Linolenic acid metabolism	C06427
Streptomycin biosynthesis	C00092
Benzoate degradation via CoA ligation	C00042

Fructose and mannose metabolism	C00392; C00794
Glycerolipid metabolism	C00093
Arginine and proline metabolism	C00327; C00624
Alanine, aspartate and glutamate metabolism	C00042
Butanoate metabolism	C00042
Citrate cycle (TCA cycle)	C00042
Propanoate metabolism	C00042
Phenylalanine, tyrosine and tryptophan biosynthesis	C00079
Glycerophospholipid metabolism	C00093
Glyoxylate and dicarboxylate metabolism	C00042
Starch and sucrose metabolism	C00092
Pentose and glucuronate interconversions	C00259
Galactose metabolism	C00794
Amino sugar and nucleotide sugar metabolism	C00140
Aminoacyl-tRNA biosynthesis	C00079

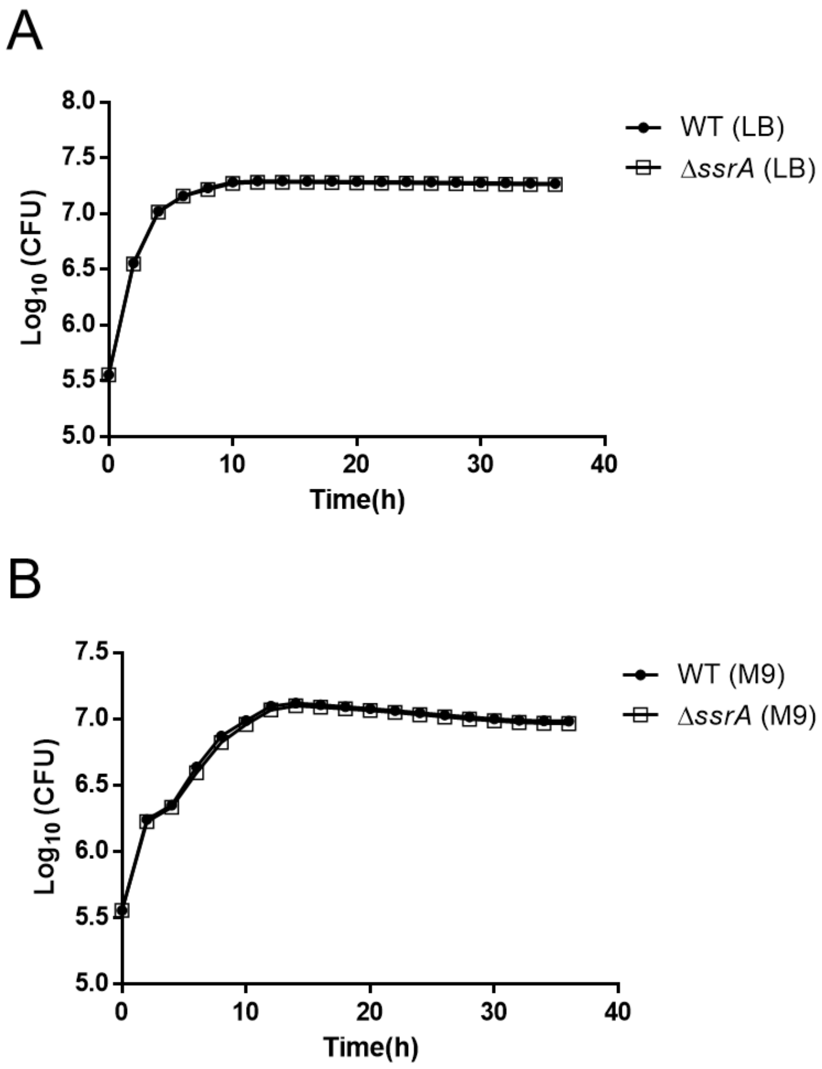

Figure S1. (A) and **(B)** WT and △*ssrA* growth curves measured by CFU counts in LB and M9 medium.

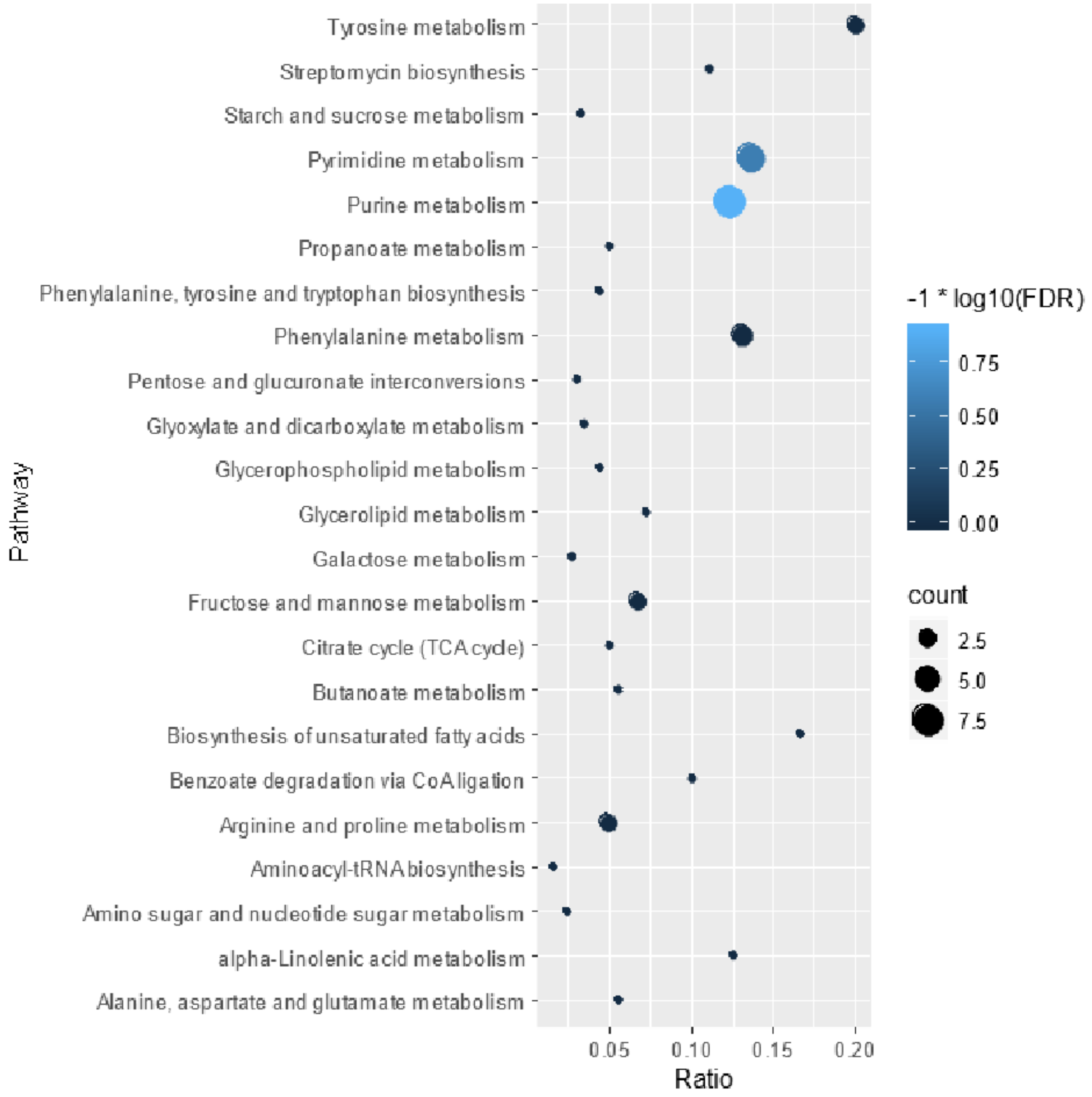

Figure S2. Bubble diagram. Pathway analysis of the essential m*e*tabolites with significant changes, wherein "FDR" refers to the P-value corrected by multiple hypo'hesis tests by the false discovery rate (FDR) method, and terms from the pathway with FDR as good or better than 1. "Ratio" refers to the ratio of number o*f th*e differential metabolites to the total number of metabolites detected in the pathway.

A

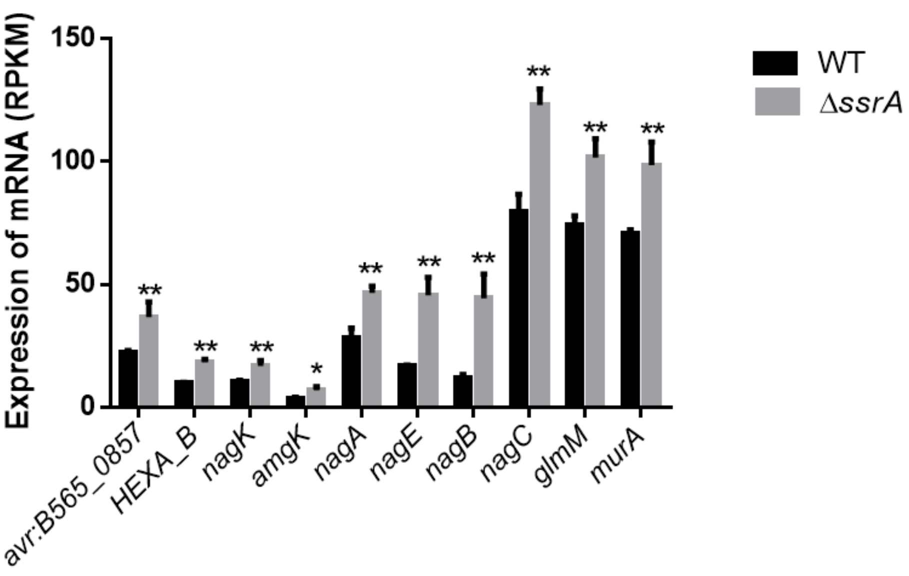

Expression of mRNA (RPKM)
150
100
50
0
WT
ΔssrA
**
*
avr:B565_0857
HEXA_B
nagK
amgK
nagA
nagE
nagB
nagC
glmM
murA

B

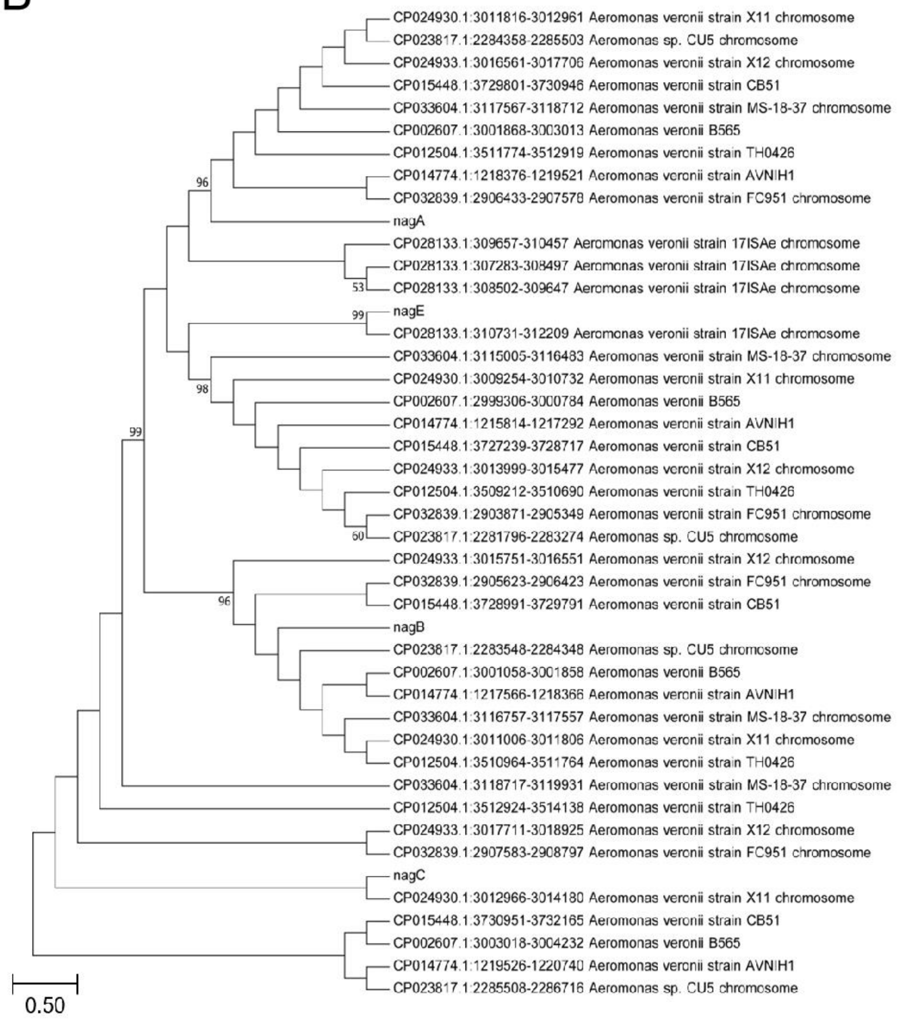

CP024930.1:3011816-3012961 Aeromonas veronii strain X11 chromosome
CP023817.1:2284358-2285503 Aeromonas sp. CU5 chromosome
CP024933.1:3016561-3017706 Aeromonas veronii strain X12 chromosome
CP015448.1:3729801-3730946 Aeromonas veronii strain CB51
CP033604.1:3117567-3118712 Aeromonas veronii strain MS-18-37 chromosome
CP002607.1:3001868-3003013 Aeromonas veronii B565
CP012504.1:3511774-3512919 Aeromonas veronii strain TH0426
CP014774.1:1218376-1219521 Aeromonas veronii strain AVNIH1
CP032839.1:2906433-2907578 Aeromonas veronii strain FC951 chromosome
nagA
CP028133.1:309657-310457 Aeromonas veronii strain 17ISAe chromosome
CP028133.1:307283-308497 Aeromonas veronii strain 17ISAe chromosome
CP028133.1:308502-309647 Aeromonas veronii strain 17ISAe chromosome
nagE
CP028133.1:310731-312209 Aeromonas veronii strain 17ISAe chromosome
CP033604.1:3115005-3116483 Aeromonas veronii strain MS-18-37 chromosome
CP024930.1:3009254-3010732 Aeromonas veronii strain X11 chromosome
CP002607.1:2999306-3000784 Aeromonas veronii B565
CP014774.1:1215814-1217292 Aeromonas veronii strain AVNIH1
CP015448.1:3727239-3728717 Aeromonas veronii strain CB51
CP024933.1:3013999-3015477 Aeromonas veronii strain X12 chromosome
CP012504.1:3509212-3510690 Aeromonas veronii strain TH0426
CP032839.1:2903871-2905349 Aeromonas veronii strain FC951 chromosome
CP023817.1:2281796-2283274 Aeromonas sp. CU5 chromosome
CP024933.1:3015751-3016551 Aeromonas veronii strain X12 chromosome
CP032839.1:2905623-2906423 Aeromonas veronii strain FC951 chromosome
CP015448.1:3728991-3729791 Aeromonas veronii strain CB51
nagB
CP023817.1:2283548-2284348 Aeromonas sp. CU5 chromosome
CP002607.1:3001058-3001858 Aeromonas veronii B565
CP014774.1:1217566-1218366 Aeromonas veronii strain AVNIH1
CP033604.1:3116757-3117557 Aeromonas veronii strain MS-18-37 chromosome
CP024930.1:3011006-3011806 Aeromonas veronii strain X11 chromosome
CP012504.1:3510964-3511764 Aeromonas veronii strain TH0426
CP033604.1:3118717-3119931 Aeromonas veronii strain MS-18-37 chromosome
CP012504.1:3512924-3514138 Aeromonas veronii strain TH0426
CP024933.1:3017711-3018925 Aeromonas veronii strain X12 chromosome
CP032839.1:2907583-2908797 Aeromonas veronii strain FC951 chromosome
nagC
CP024930.1:3012966-3014180 Aeromonas veronii strain X11 chromosome
CP015448.1:3730951-3732165 Aeromonas veronii strain CB51
CP002607.1:3003018-3004232 Aeromonas veronii B565
CP014774.1:1219526-1220740 Aeromonas veronii strain AVNIH1
CP023817.1:2285508-2286716 Aeromonas sp. CU5 chromosome
96
53
99
98
99
96
0.50

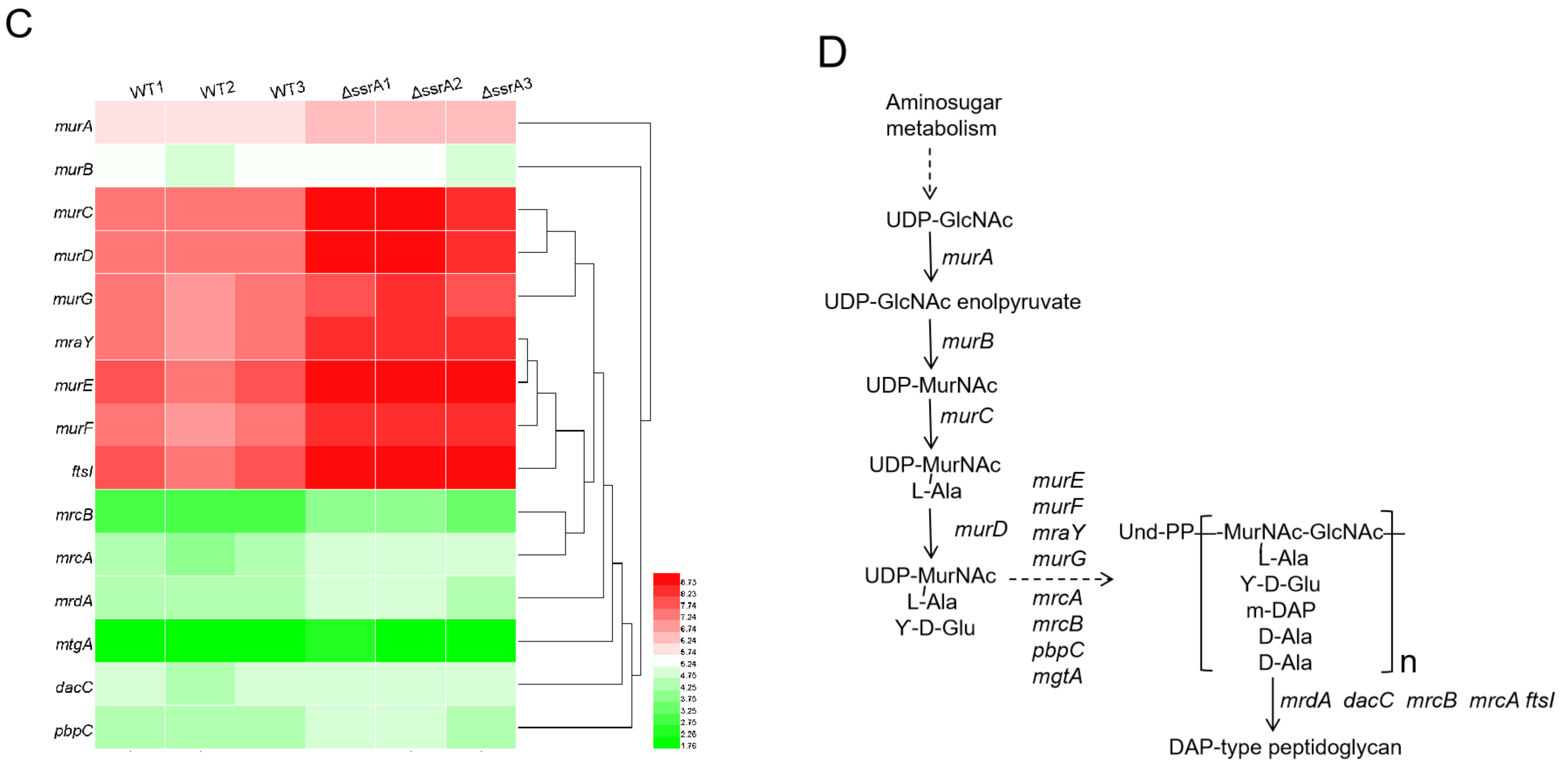

Figure S3. (A) The expression levels of the genes associated with the metabolism of GlcNAc in WT and △*ssrA* strains, which were revealed in transcriptomic analysis. * indicates a significant difference with $p < 0.05$, and ** indicates a greatly significant result with $p < 0.01$, followed by one-way ANOVA, Turkey post-test. **(B)** The phylogenetic tree of *nagA*, *nagB*, *nagC*, *nagE* in the genus *Aeromonas*, wherein the values on the branches indicate the reliability of this branch. **(C)** Heat map showing the gene expression profiles of peptidoglycan synthesis in WT and △*ssrA* strains. **(D)** Peptidoglycan biosynthesis pathway. Italic font represents a gene, and the arrows indicate the direction of the metabolic flux.

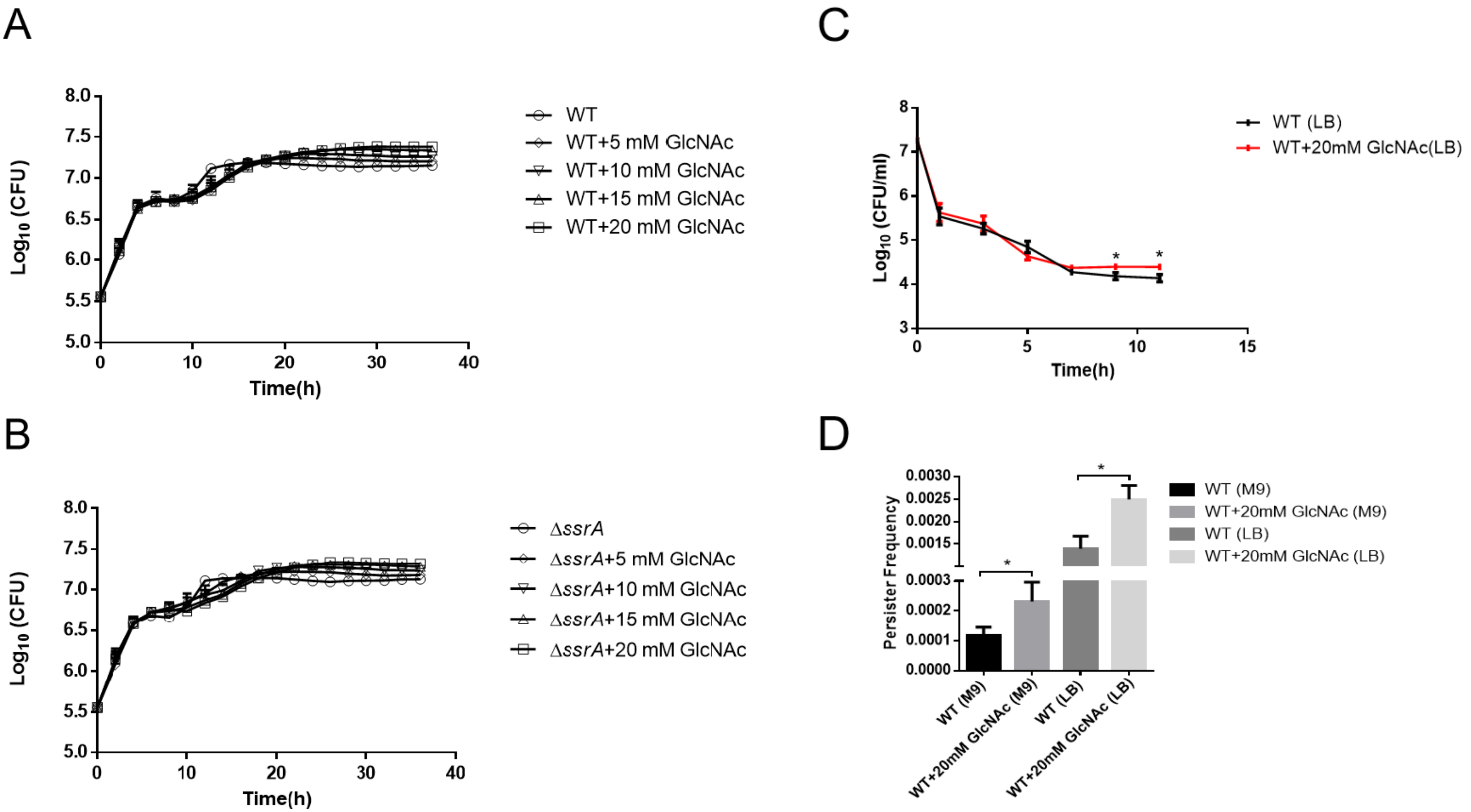

Figure S4. (A) and **(B)** Growth curves measured by CFU counts for WT and △*ssrA* strain supplemented without and (WT/△*ssrA*) with 5 mM GlcNAc (5 mM GlcNAc+WT/△*ssrA*), 10 mM GlcNAc (10 mM GlcNAc+WT/△*ssrA*), 15 mM GlcNAc (15 mM GlcNAc+WT/△*ssrA*), and 20mM GlcNAc (20 mM GlcNAc+WT/△*ssrA*) in M9 medium. **(C)** and **(D)** Bacterial survival curve and persister frequency showing the persister formations in WT in the absence and presence of 20 mM GlcNAc in LB medium under the treatment with cefotaxime for 11 hours. * indicates a significant result with $p < 0.05$, followed by one-way ANOVA, Turkey post-test.
